# p.N370S *GBA1* Mutation Influences the Morphology and Lipid Composition of Extracellular Vesicles in Blood Plasma from Patients with Parkinson’s Disease

**DOI:** 10.3390/ijms26189152

**Published:** 2025-09-19

**Authors:** Tatiana S. Usenko, Alena E. Kopytova, Artem D. Izyumchenko, Darya G. Kulabukhova, Artemiy S. Silantyev, Victoria D. Kazakova, Katerina S. Basharova, Anastasia I. Bezrukova, Luiza A. Garaeva, Evgeny B. Pichkur, Alexandra V. Artynyuk, Irina V. Miliukhina, Alla A. Timofeeva, Valentina V. Miroshnikova, Stanislav N. Naryzhny, Anton K. Emelyanov, Natalya B. Zakharzhevskaya, Andrey L. Konevega, Tatiana A. Shtam, Sofya N. Pchelina

**Affiliations:** 1 Petersburg Nuclear Physics Institute Named by B.P. Konstantinov of National Research Centre «Kurchatov Institute», Gatchina 188300, Russia; usenko_ts@pnpi.nrcki.ru (T.S.U.); kopytova_ae@pnpi.nrcki.ru (A.E.K.); izyumchenko_ad@pnpi.nrcki.ru (A.D.I.); kulabuhovadarya@gmail.com (D.G.K.); basharova_ks@pnpi.nrcki.ru (K.S.B.); bezrukova_ai@pnpi.nrcki.ru (A.I.B.); garaeva_laa@pnpi.nrcki.ru (L.A.G.); pichkur_eb@nrcki.ru (E.B.P.); aleksandra.artynyuk@gmail.com (A.V.A.); miroshnikova_vv@pnpi.nrcki.ru (V.V.M.); naryzhnyy_sn@pnpi.nrcki.ru (S.N.N.); emelyanov_ak@pnpi.nrcki.ru (A.K.E.); konevega_al@pnpi.nrcki.ru (A.L.K.); shtam_ta@pnpi.nrcki.ru (T.A.S.); 2 Department of Molecular Genetic and Nanobiological Technologies, Pavlov First Saint Petersburg State Medical University, St. Petersburg 197022, Russia; timmma@mail.ru; 3 Lopukhin Federal Research and Clinical Center of Physical-Chemical Medicine of Federal Medical Biological Agency, Moscow 143007, Russia; artsilan@gmail.com (A.S.S.); Viktorialuzanova.1998@gmail.com (V.D.K.); natazaha@gmail.com (N.B.Z.); 4 National Research Center “Kurchatov Institute”, Moscow 123098, Russia; 5 N.P. Bechtereva Institute of the Human Brain of Russian Academy of Sciences, St. Petersburg 197022, Russia; milyukhinaiv@yandex.ru

**Keywords:** Parkinson’s disease, *GBA1*, extracellular vesicles, cryo-electron microscopy, lipidomics, proteomics

## Abstract

Parkinson’s disease, associated with mutations in the *GBA1* gene (GBA1-PD), is the most common genetic form of Parkinson’s disease (PD), marked by clinical heterogeneity influenced by mutation type. Extracellular vesicles (EVs), key mediators of intercellular communication, are implicated in PD pathogenesis through the transport of pathological proteins and lipids. In this study, we analyzed blood plasma-derived EVs from GBA1-PD patients carrying p.N370S and p.L444P mutations and from healthy controls using cryo-electron microscopy, lipidomics, and proteomics. EVs from GBA1-PD patients were significantly larger than those from controls, with the largest size and most multilayered vesicles observed in p.N370S carriers. Lipidomic profiling identified 237 lipid species; of these, 186 lipids were altered in p.N370S and 24 in p.L444P versus controls. Mutation carriers showed distinct lipid signatures, with p.L444P samples enriched predominantly in sphingolipids, while p.N370S carriers exhibited more extensive lipid remodeling across multiple classes, including triglycerides, cholesteryl esters, and phospholipids. Notably, Cer 23:0 was elevated across all GBA1-PD groups. Proteomic analysis revealed enrichment in pathways related to lipid transport, immune regulation, and vesicle-mediated processes. Overall, GBA1-PD patients share a distinct lipidomic EV signature, with mutation-specific patterns reflecting differing mechanisms of lysosomal dysfunction. These findings support the potential of EV profiling to unravel disease heterogeneity and identify biomarkers.

## 1. Introduction

Extracellular vesicles (EVs), including exosomes, are membrane-bound nanoparticles secreted by most cell types. These vesicles play a crucial role in intercellular communication by transporting proteins, lipids, and nucleic acids, and are involved in a wide range of physiological and pathological processes [1]. Alterations in EV size, morphology, and molecular cargo have been associated with various diseases, including cancer, metabolic disorders, and neurodegenerative diseases.

In recent years, increasing attention has been given to the role of EVs in Parkinson’s disease (PD)—a progressive neurodegenerative disorder affecting 1–2% of individuals over 60 years of age. PD is characterized by the selective loss of dopaminergic neurons in the substantia nigra and the accumulation of misfolded alpha-synuclein, forming intracellular aggregates known as Lewy bodies, a pathological hallmark of the disease. Importantly, EVs have been implicated in the propagation of alpha-synuclein pathology. Several studies have suggested that EVs can transport aggregated alpha-synuclein between neurons, thereby contributing to the spreading of neurodegeneration across different brain regions [2,3,4,5]. Moreover, several studies have suggested that alpha-synuclein levels in EVs derived from different biological fluids (blood plasma, cerebrospinal fluid (CSF), saliva) could serve as disease biomarkers [6,7,8]. It has been suggested that the lipid composition of EV membranes might influence alpha-synuclein oligomerization, as several classes of lipids have been shown to induce alpha-synuclein seeding in vitro [9,10,11].

While the majority of PD cases are sporadic, a significant subset is linked to mutations in the specific genes. Among them, mutations in the *GBA1* gene—encoding the lysosomal enzyme β-glucocerebrosidase (GCase)—represent the most common genetic risk factor for PD [12,13,14,15,16]. Biallelic *GBA1* mutations cause Gaucher disease (GD), a lysosomal storage disorder (LSD) characterized by impaired degradation of glucosylceramide and glucosylsphingosine, resulting in lipid accumulation within lysosomes [17]. We have previously shown that in patients with GD, plasma EVs exhibit altered morphology and size [18], suggesting that lysosomal dysfunction and disrupted lipid metabolism may influence EV biogenesis and secretion. These findings suggest a possible link between LSDs, EV dysregulation, and neudegeneration.

It is important to note that *GBA1* mutations differ in severity and their impact on phenotypes. “Severe” mutations (such as p.L444P) are generally associated with more aggressive forms of GD and earlier, more severe PD manifestations, whereas “mild” mutations (such as p.N370S) tend to result in milder GD phenotypes and variable PD progression [19,20]. This genotype–phenotype correlation highlights the heterogeneity in clinical presentation and disease progression among *GBA1* mutation carriers.

Of note, even heterozygous *GBA1* mutations, while not sufficient to cause GD, significantly increase the risk (by 5–7-fold) of developing PD. These GBA1-associated PD (GBA1-PD) cases account for ~10% of all PD and are characterized by earlier onset, faster progression, and more severe cognitive decline [12,13,14,15,16]. Although the precise mechanisms linking reduced GCase activity due to genetic mutations in the *GBA1* gene to PD remain unclear, a widely accepted hypothesis proposes that impaired lysosomal degradation of alpha-synuclein leads to its accumulation [21]. Reduced activity of GCase through genetic or chemical means leads to an increase in EV release in both cellular and animal studies [22,23,24,25], suggesting that EV-mediated mechanisms may play a central role in the GBA1-PD pathogenesis.

In the current study, we examined how *GBA1* mutations may affect the structure and molecular cargo of blood plasma EVs in PD patients. We combined cryo-electron microscopy (cryo-EM), lipidomic assays, and proteomic assays to characterize plasma EVs derived from GBA1-PD patients bearing different mutations in the *GBA1* gene (p.N370S and p.L444P).

## 2. Results

**Figure 1:**
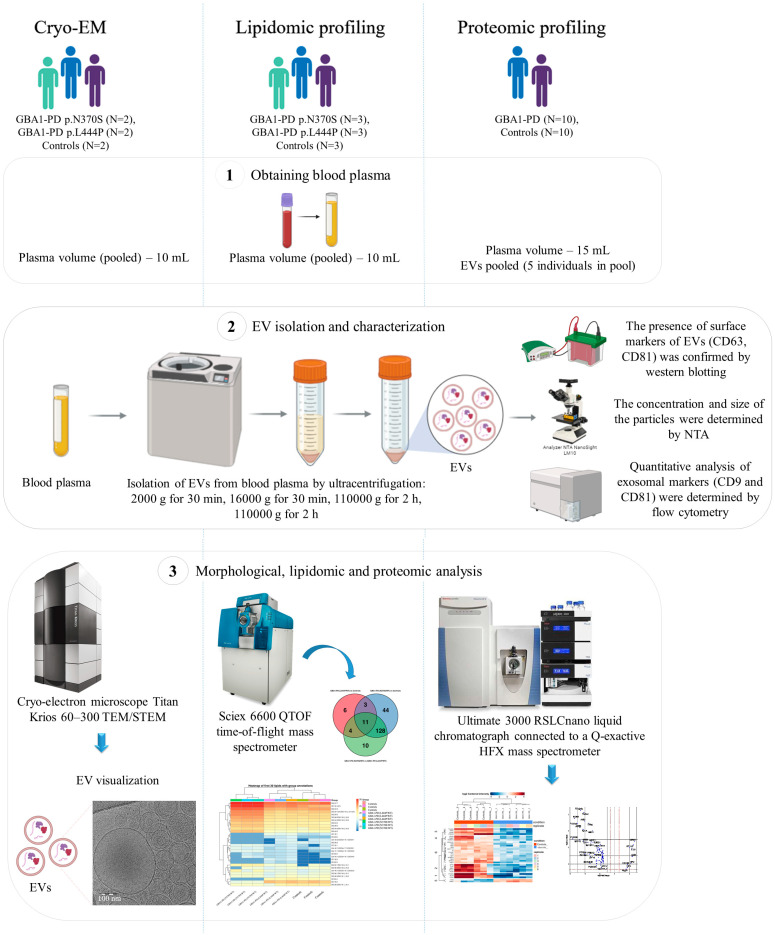
Schematic overview of the experimental workflow. Blood plasma samples were collected from GBA1-PD patients carrying either the p.N370S or p.L444P mutation and from controls. A total of 11 GBA1-PD patients and 15 controls were included in the study. EVs were isolated and characterized using a multi-modal approach combining nanoparticle tracking analysis (NTA), Western blot, flow cytometry, cryo-EM, lipidomic profiling, and proteomic analysis. Cryo-EM was used to assess EV morphology and size distribution. Lipidomic analysis was performed to identify and quantify lipid species within EVs, while proteomic profiling provided insights into protein cargo and related functional pathways. Created with BioRender.com (accessed on 6 August 2025).

To investigate EV alterations associated with GBA1-PD, we isolated blood plasma EVs from patients carrying heterozygous p.N370S or p.L444P mutations and from the matched healthy controls. Samples were analyzed using a multi-modal approach, including flow cytometry and Western blot for EV marker validation, nanoparticle tracking analysis (NTA), and cryo-EM for morphological analysis, as well as lipidomic and proteomic profiling. A schematic overview of the experimental workflow is shown in Figure 1. Importantly, pooled and non-pooled EV samples were used across different experiments to account for variability and confirm findings across sample types. Key experimental steps and sample grouping are indicated in the schematic.

### 2.1. Morphological Characterization of EVs by Cryo-EM

**Figure 2:**
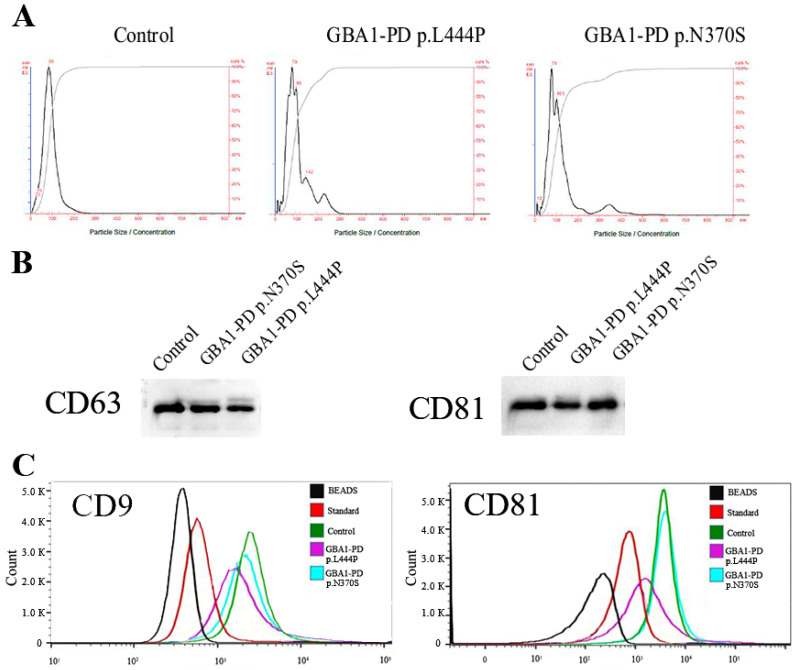
Characterization of EVs from the blood plasma of GBA1-PD patients and controls by NTA, Western blot, and flow cytometry. (**A**) Quantification of vesicular size and concentration by NTA. (**B**) Western blot analysis of the CD63 and CD81 as common exosomal markers in several samples of isolated EVs from plasma of GBA1-PD patients and controls. (**C**) Example of flow cytometric analysis of the CD9 and CD81 exosomal marker expression on the surface of EVs isolated from the blood plasma. Immunobeads, which were not incubated with EVs during sample preparation, were used as a negative control (BEADS). The exosomal standard included in the HansaBioMed exosome cytometric assay kit was used as a positive control (standard).

EVs were isolated from 10 mL of pooled blood plasma (2 individuals in each group (GBA1-PD p.N370S, GBA1-PD p.L444P, and controls)) using ultracentrifugation according to the EV purification protocol previously described [18]. Characterization of EVs was performed in accordance with International Society for Extracellular Vesicles guidelines [1]. EVs were validated by NTA (Figure 2A). In the samples from GBA1-PD patients with p.N370S mutation in the *GBA1* gene, the measured parameter D90 (the diameter at which 90% of the sample’s mass comprises particles with a diameter less than this value) and the size mode of EVs were 162.7 ± 30.2 nm and 81.0 ± 6.2 nm, with the concentration ranging from 1.8 × 10^13^ to 2.8 × 10^13^ particles/mL, respectively. In the samples from GBA1-PD patients with p.L444P mutation in the *GBA1* gene, these values were 199.0 ± 29.5 nm and 77.7 ± 11.1 nm, with the concentration ranging from 1.6 × 10^13^ to 2.4 × 10^13^ particles/mL, respectively. In the samples from the control group, parameter D90 and the size mode of EVs were 154.7 ± 20.5 nm and 87.0 ± 5.6 nm, with the concentration ranging from 2.1 × 10^13^ to 3.9 × 10^13^ particles/mL, respectively. The presence of exosomal marker tetraspanin CD81 and CD63, as well as CD9 and CD81, was confirmed by Western blot and flow cytometry in all samples, respectively (Figure 2B,C).

Morphological characteristics of EVs were performed via cryo-EM in GBA1-PD patients and controls. Cryo-EM allowed us to visualize EVs of various sizes and morphologies with lipid bilayers and vesicle internal structures. Most of the vesicles were intact and had a round shape. In total, images of 240 particles were analyzed for plasma samples from GBA1-PD (p.N370S), with 645 particles for GBA1-PD (p.L444P) patients and 89 particles for the samples from individuals of the comparison group.

**Figure 3:**
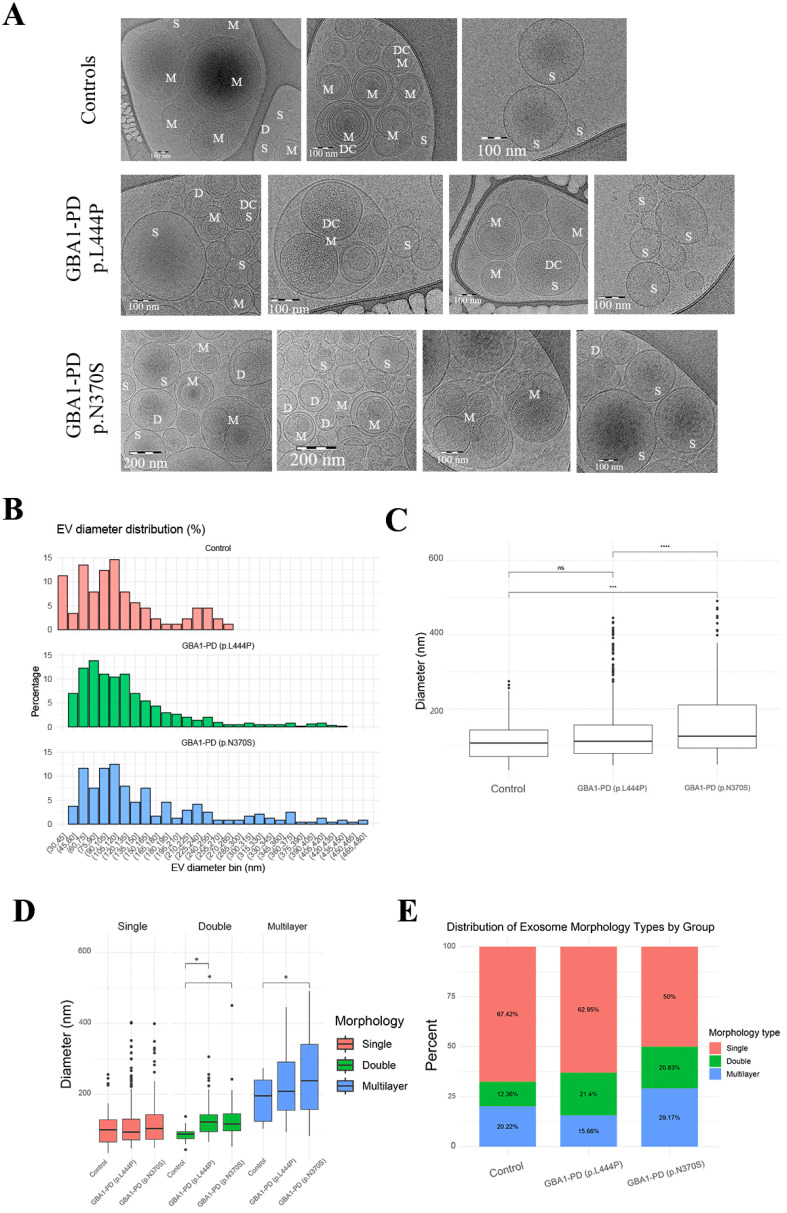
Characterization of EVs by cryo-EM. (**A**) Cryo-EM images of EVs isolated from the blood plasma of controls and GBA1-PD. Various morphological types of EVs have been identified: single vesicles (S), double vesicles (D), multilayered vesicles (M), and vesicles with electron-dense cargo (DC). (**B**) Size distribution of EVs in controls and GBA1-PD patients carrying p.L444P or p.N370S mutations. EV diameters were binned in 15 nm intervals, and the percentage of EVs within each bin is shown. Panels are stacked vertically by group: control—red, GBA1-PD (p.L444P)—green, and GBA1-PD (p.N370S)—blue. (**C**) Representation in size of EVs isolated from the blood plasma of controls and GBA1-PD. (**D**) Representation in size of EVs isolated from the blood plasma of controls and GBA1-PD with various morphologies (single (red), double (green) and multilayer vesicles (blue)). (**E**) Proportional distribution diagram for main types of observed EVs. Single (red), double (green), and multilayer vesicles (blue). Symbols indicate significance levels: * *p* < 0.05, *** *p* < 0.001, **** *p* < 0.0001; ns, not significant.

Most of the vesicles were intact and had a round shape. When averaged, plasma EVs in GBA1-PD were characterized by a larger size, 116.6 (48.4–491.3) nm, compared to those in controls 108.0 (35.0–274.6) nm (*p* = 0.033). Interestingly, plasma EVs in GBA1-PD (p.N370S) were characterized by a larger size, 126.5 (49.9–491.3) nm, compared to those in GBA1-PD (p.L444P), 112.7 (48.4–445.7) nm, and controls (*p* < 0.0001 and *p* < 0.001, respectively) (Figure 3B,C).

Visualized particles could be classified as single, double, multilayer, and vesicles with electron-dense cargo (Figure 3A). Single particles prevailed in EV samples (67.4% for controls, 59.4% for GBA1-PD, 62.9% for GBA1-PD (p.L444P), and 50% for GBA1-PD (p.N370S)) (Figure 3E). Pairwise group comparisons using Fisher’s exact test revealed a highly significant difference in the morphology of EVs between patients with GBA1-PD carrying the p.L444P and p.N370S mutations (*p* = 1.37 × 10^−9^). The difference between GBA1-PD patients carrying the p.L444P and controls did not reach statistical significance (*p* = 0.088), whereas the difference between GBA1-PD patients carrying the p.N370S carriers and controls was significant (*p* = 0.0108) (Figure 3E).

When dividing patients by the type of mutation in the *GBA1* gene, we found that in samples from GBA1-PD (p.N370S) patients, the EV diameters of double vesicles, 116.8 (53.3–450.5) nm, and multilayer, 219.0 (83.0–491.3) nm, vesicles were larger compared to those in the control group, 88.4 (44.4–138.0) nm and 196.3 (103.7–274.6) nm (*p* = 0.0029 and *p* = 0.024, respectively) (Figure 3D). Also, the size of double particles of GBA1-PD (p.L444P), 122.5 (66.3–306.5) nm, was larger compared to those in the control group (*p* = 0.002) (Figure 3D).

It is interesting to note that despite the absence of a statistically significant difference in the diameter of single EVs between the studied groups, a lot of single EVs with a large size (131.5–402.9 nm) were observed in GBA1-PD groupm both in the carriers of p.N370S and p.L444P mutations (Figure 3C). Moreover, the single EVs with a diameter over 256.4 nm were detected in GBA1-PD but were absent in the controls.

### 2.2. Lipidomic Profiling of EVs from GBA1-PD Patients Compared to Controls with Stratification by Mutation

EVs for lipidomic profiling were isolated from 10 mL of pooled plasma (3 individuals in each group (GBA1-PD p.N370S, GBA1-PD p.L444P, and controls) using ultracentrifugation according to the EV purification protocol.

**Figure 4:**
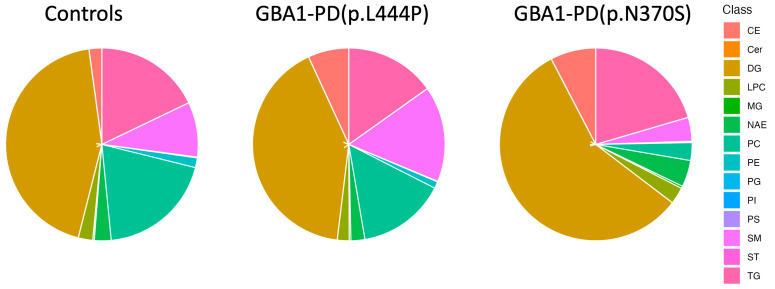
Proportional distribution of lipid classes of plasma EVs across sample groups: controls, GBA1-PD patients with the p.L444P mutation, and GBA1-PD patients with the p.N370S mutation. The pie chart illustrates differences in lipid composition among these cohorts.

**Figure 5:**
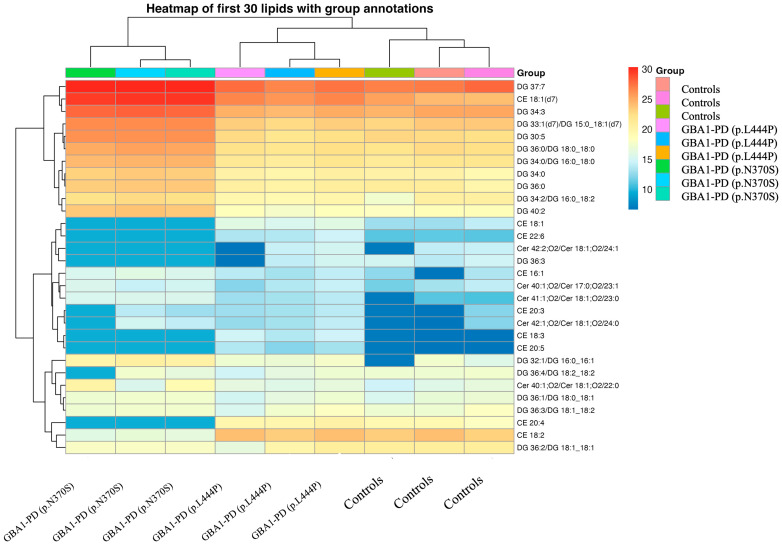
Heatmap showing differential lipid expression in plasma EVs between GBA1-PD patients and controls. Rows represent individual lipids; columns represent samples. Colors indicate relative expression (blue = low, red = high).

An untargeted lipidomic analysis was performed on EVs isolated from blood plasma using an Exion 30AD liquid chromatograph and a Sciex 6600 QTOF time-of-flight mass spectrometer equipped with a calibrant delivery system (CDS). In total, 237 lipid species were reliably quantified across the main lipid classes, including sterol lipids, glycerolipids, sphingolipids, glycerophospholipids, and lysophospholipids, in all study groups (GBA1-PD patients and controls). The dataset included biologically relevant lipid subclasses such as cholesteryl esters (CE), ceramides (Cer), diacylglycerols (DGs), monoacylglycerols (MGs), lysophosphatidylcholines (LPCs), phosphatidylcholines (PCs), phosphatidylinositols (PIs), phosphatidylserines (PSs), phosphatidylethanolamines (PEs), N-acylethanolamines, sphingomyelins (SMs), and triacylglycerols (TGs), among others. A pie chart illustrates the distribution of lipid classes in plasma EVs from GBA1-PD patients and controls (Figure 4). All groups showed a predominance of DGs. Notably, GBA1-PD patients carrying p.L444P mutation exhibited lower DG levels alongside relatively higher proportions of PCs and SMs compared to p.N370S mutation carriers (Figure 4). The heatmap highlights the most significant differences in lipidomic profiles between GBA1-PD patients and controls, with lipids organized by class—beginning with DGs, followed by CEs, and Cer (Figure 5). Importantly, the lipid composition in p.L444P mutation carriers appeared more similar to controls, with only moderate alterations, whereas p.N370S mutation carriers showed a markedly different lipid profile characterized by broader and more pronounced lipid dysregulation across multiple lipid classes.

**Figure 6:**
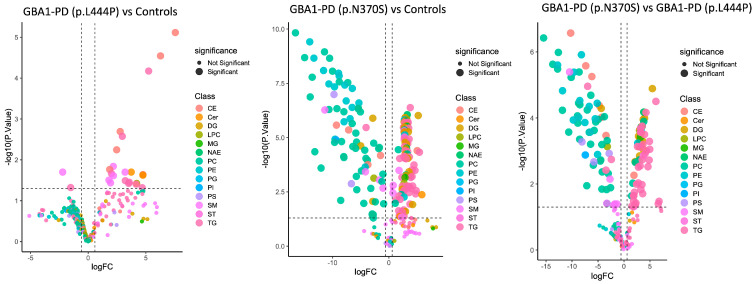
Volcano plot for EV lipids between the studied groups; the upregulated and downregulated lipids with *p* < 0.05 and |FC| > 1.5. The *x*-axis corresponds to the log2 fold change (FC), and the *y*-axis to the –log10 *p*-value, reflecting both the magnitude and statistical significance of the observed differences. Lipids with *p* < 0.05 and |FC| > 1.5 are highlighted as significantly upregulated (right side) or downregulated (left side). The vertical dashed lines indicate the fold change thresholds (log2 FC = ±1.5), while the horizontal dashed line corresponds to the statistical significance threshold (*p* = 0.05). These thresholds were applied to identify biologically relevant lipid alterations across the studied groups.

Next, we conducted comparative lipidome analysis of EVs between studied groups. A total of 62 differentially expressed lipids were identified in blood plasma EVs of GBA1-PD patients compared to controls, with 28 lipids showing increased expression and 24 showing decreased expression (*p* < 0.05 and |FC| > 1.5) (Figure 6).

We also conducted a comparative lipidomic analysis of blood plasma EVs from GBA1-PD patients carrying the p.L444P mutation and controls, which revealed 24 differentially expressed lipids—twenty-two upregulated and two downregulated (*p* < 0.05, |FC| > 1.5). In a similar analysis of EVs from GBA1-PD patients with the p.N370S mutation, 186 lipids were found to be differentially expressed, including 108 upregulated and 78 downregulated species. Furthermore, a comparison between p.L444P and p.N370S mutation groups revealed 153 differentially expressed lipids, with 83 decreased and 70 increased (Figure 5, Supplementary Table S1).

**Figure 7:**
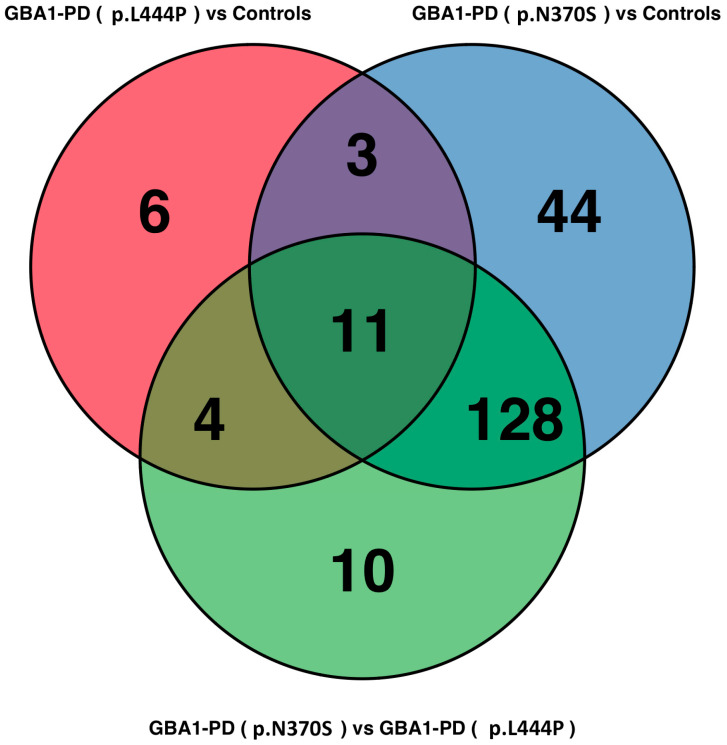
Venn diagram showing the overlap of differentially abundant EV lipid species between GBA1-PD subgroups (p.L444P and p.N370S) and neurologically healthy controls. The overlapping areas indicate shared lipid alterations across comparisons, while non-overlapping segments reflect mutation-specific lipid changes. The results highlight distinct and common lipid dysregulation patterns in GBA1-PD subtypes.

Additionally, in the current study, we analyzed differentially abundant EV lipids across three pairwise comparisons, GBA1-PD (p.L444P) vs. controls, GBA1-PD (p.N370S) vs. controls, and GBA1-PD (p.N370S) vs. GBA1-PD (p.L444P), using thresholds of log2 fold change > log2(1.5) and *p*-value < 0.05. In total, 14 lipids were commonly altered between both GBA1-PD patients and controls, of which 11 were shared across all three comparisons, including GBA1-PD (p.N370S) vs. GBA1-PD (p.L444P) (Figure 7, Supplementary Table S2). These consistently dysregulated lipids predominantly belonged to three main lipid classes: CEs, TGs, and sphingolipids (including Cer and SMs). Specifically, altered cholesteryl esters (CE 18:1, CE 18:3, CE 20:5, CE 22:6), multiple triglyceride species (TG 52:5, TG 54:3, TG 56:1., etc.), and sphingolipids (Cer 23:1 and SM 41:3; O2) highlight possible significant perturbations in cholesterol metabolism, neutral lipid storage, and sphingolipid homeostasis across GBA1-PD regardless of mutation type. Interestingly, six lipids were uniquely altered in the GBA1-PD (p.L444P) group, mainly involving sphingomyelins (SM 35:1; O2, SM 34:2; O2), ceramides (Cer 24:0), and selected triglycerides and cholesteryl esters, suggesting a more specific remodeling of sphingolipid metabolism in p.L444P *GBA1* mutation carriers. Notably, Cer 24:0 accumulation appeared exclusive to p.L444P mutation carriers, whereas Cer 23:0 was elevated across all GBA1-PD groups, albeit at higher levels in p.N370S individuals. Strikingly, GBA1-PD (p.N370S) patients demonstrated the most extensive lipid alterations, with 128 lipids significantly differing from both controls and p.L444P carriers. Class-specific analysis revealed that these changes spanned a broad spectrum of lipid types, with a pronounced enrichment in triglycerides and cholesteryl esters, accompanied by marked disturbances in sphingolipids (Cer, SM) and phospholipids, including PCs, PEs, and plasmalogens. This suggests that the p.N370S mutation may be associated with more profound disruptions in energy metabolism, cholesterol handling, and membrane lipid remodeling, including pathways related to mitochondrial function and oxidative stress defense. Overall, these findings indicate a shared EV lipidomic signature in GBA1-PD, predominantly driven by disturbances in neutral lipids and sphingolipids, with mutation-specific patterns revealing a broader and more severe lipid remodeling in p.N370S carriers and a more targeted sphingolipid dysregulation in p.L444P carriers.

### 2.3. Proteomic Profiling of EVs from GBA1-PD Patients Compared to Controls

**Figure 8:**
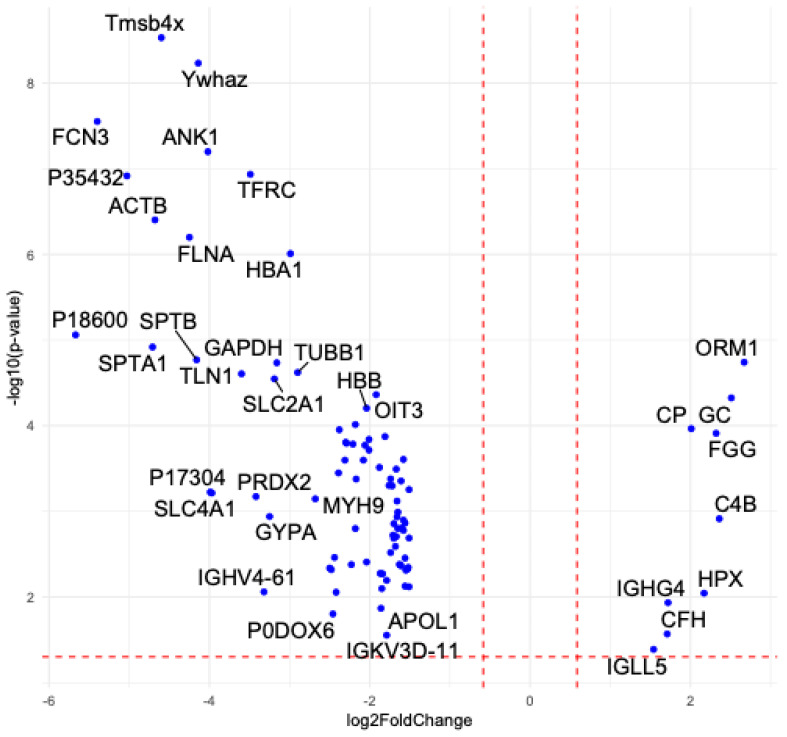
Volcano plot of EV protein expression changes in GBA1-PD versus controls. Proteins with *p* < 0.05 and |log2 fold change| > 1.5 are highlighted. Each point corresponds to an individual protein. The *x*-axis shows the log2 fold change, and the *y*-axis shows the –log10 *p*-value. Proteins with *p* < 0.05 and |log2 fold change| > 1.5 are highlighted as significantly upregulated or downregulated. The vertical dashed lines indicate the fold change thresholds (log2 FC = ±1.5), and the horizontal dashed line marks the significance threshold (*p* = 0.05).

**Figure 9:**
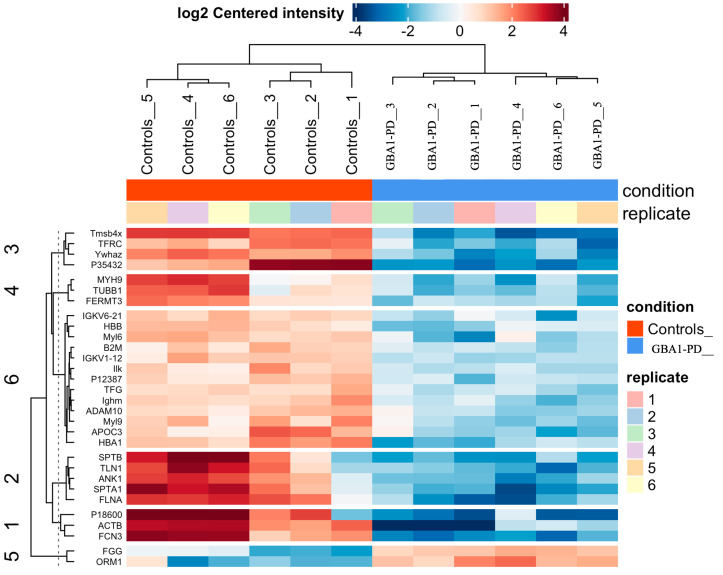
Heatmap showing differential expression of EV proteins between GBA1-PD patients and controls. Rows represent individual proteins; columns represent samples. Colors indicate relative expression (blue = low; red = high).

EVs for proteomic profiling were isolated from 15 mL of plasma (10 individuals in each group (GBA1-PD without division by type of mutation in the *GBA1* gene and controls)) using ultracentrifugation separately for each individual according to the EV purification protocol, and then EV samples were pooled from each five individuals per one pool. To investigate molecular alterations in EVs associated with GBA1-PD, we performed proteomic profiling of blood plasma EVs from GBA1-PD patients as well as healthy controls. A total of 262 EV proteins were identified, of which 91 were differentially expressed (*p* < 0.05, |log2 FC| > 1.5). Strikingly, eighty-two proteins were significantly downregulated in GBA1-PD compared to controls, while only nine showed upregulation (Figure 8, Supplementary Table S3). The heatmap of differentially expressed proteins clearly demonstrates a consistent downregulation pattern across the GBA1-PD group relative to controls, suggesting global suppression of key EV-associated proteins, particularly those involved in lipid transport and vesicle-mediated processes (Figure 9).

**Figure 10:**
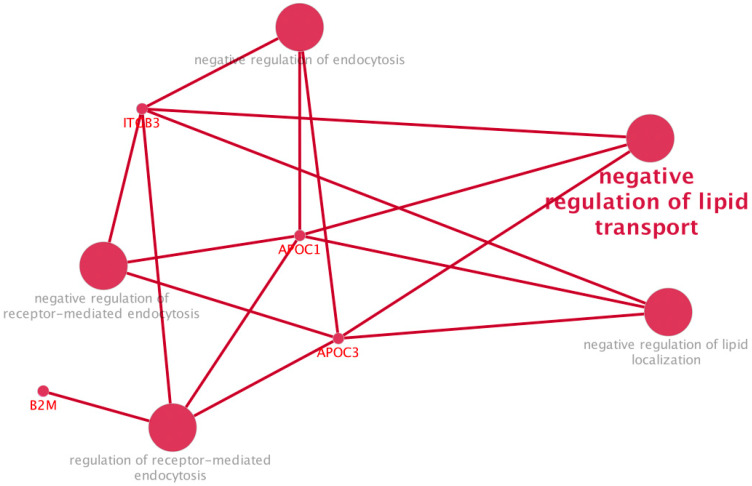
Functional enrichment analysis of differentially expressed EV proteins in GBA1-PD patients compared to controls.

Functional enrichment analysis of these differentially expressed proteins using ClueGO (Cytoscape plugin, version 2.5.10, https://apps.cytoscape.org/apps/cluego, accessed on 28 February 2025), focusing on GO Biological Process terms, highlighted a strong overrepresentation of processes associated with lipid transport and vesicle-mediated exchange, particularly negative regulation of lipid transport (GO:0032369), negative regulation of lipid localization (GO:1905953), negative regulation of endocytosis (GO:0045806), and negative regulation of receptor-mediated endocytosis (GO:0048261) (Figure 10). These pathways point toward a disrupted ability of EVs to properly deliver lipid cargo or be internalized by target cells, which may have significant implications in the context of PD pathogenesis. Apolipoproteins such as APOC1 and APOC3, both known to inhibit lipoprotein receptor binding and regulate lipid transfer efficiency, were prominently involved in these processes and significantly downregulated in GBA1-PD EVs.

Beyond lipid-related pathways, the proteomic enrichment analysis of EVs from GBA1-PD patients also highlighted several additional biological processes of interest (Supplementary Figure S1, Supplementary Table S4). These include immune-related responses such as the humoral immune response (GO:0006959), complement activation (GO:0006956), opsonization (GO:0008228), and phagocytosis recognition (GO:0006910), indicating potential immunomodulatory roles of EVs in the context of GBA1-PD. Several terms related to oxidative stress and antioxidant defense were also enriched, including hydrogen peroxide catabolism (GO:0042744), oxidant detoxification (GO:0098869), and peroxidase activity (GO:0004601), reflecting an upregulation of redox-related processes. This aligns with the known vulnerability of dopaminergic neurons to oxidative damage in PD and further implicates EVs as potential conveyors or modulators of oxidative burden. Additionally, processes such as retina homeostasis (GO:0001895), blood–brain barrier maintenance (GO:0035633), focal adhesion assembly (GO:0048041), and protein localization to cell–cell junction (GO:0150105) suggest a broader role for EVs in maintaining tissue integrity and intercellular communication, both of which are disrupted in neurodegenerative diseases. Finally, the analysis identified pathways related to platelet activation and coagulation (platelet aggregation (GO:0070527), hemostasis (GO:0007599), blood coagulation, and fibrin clot formation (GO:0072378)).

## 3. Discussion

In the current study, we present a comprehensive characterization of plasma-derived EVs from GBA1-PD patients, integrating morphological, lipidomic, and proteomic analyses. For the first time, we directly compared EV profiles between GBA1-PD patients carrying different mutations. Our findings reveal that EVs from GBA1-PD patients exhibit distinct structural and molecular features compared to healthy controls, with clear mutation-specific differences between p.N370S and p.L444P carriers. Notably, EVs from p.N370S patients showed the most pronounced alterations, including increased vesicle size, a higher proportion of double and multilayered vesicles, and extensive lipidome remodeling. In contrast, EVs from p.L444P carriers displayed more modest lipidomic changes overall but showed a focused increase in sphingolipids, particularly ceramides with long acyl chains, consistent with mutation-specific dysregulation of sphingolipid metabolism.

EVs are nanoscale membrane-bound particles secreted by nearly all cell types. They play a key role in intercellular communication through the transport of proteins, lipids, and nucleic acids. Several studies regarding neurodegenerative disorders aim to evaluate the potential of EVs in different biofluids as biomarkers [26]. Among these, CSF is more challenging for patients to provide compared to other more accessible biofluids such as blood, urine, and saliva. Numerous studies have investigated EVs of various origins in the pathogenesis of PD [27,28]. Some studies have reported no significant differences in the concentration of EVs in plasma/serum or urine between PD patients and healthy controls [29,30,31]. Stuendl et al. observed a reduction in the number of plasma-derived EVs in individuals with PD [32]. In contrast to these findings, our previous work demonstrated an increased concentration of EVs in the blood plasma of patients with PD and GBA1-associated PD [33]. Additionally, elevated levels of saliva-derived EVs were detected in PD patients [34]. Among the various EV subtypes, exosomes (30–150 nm) originate from the inward budding of endosomal membranes, forming multivesicular bodies (MVBs) that can either fuse with lysosomes for degradation or with the plasma membrane to release their intraluminal vesicles as exosomes into the extracellular space [35]. This decision point is not yet fully understood but appears to be regulated by lysosomal function [36].

Indeed, lysosomal dysfunction is increasingly recognized as a trigger for altered EV release. When lysosomal degradation is impaired, cells may redirect MVBs toward the plasma membrane, thereby enhancing exosome secretion as a compensatory mechanism [35]. Supporting this model, pharmacological inhibition of lysosomal acidification with bafilomycin A1 increases EV release and promotes the secretion and transmission of alpha-synuclein—a key protein in PD pathogenesis—via exosomes [37,38]. Similarly, Eitan and colleagues demonstrated that lysosomal dysfunction enhances EV production across various models [36]. Alterations in EV morphology were previously observed in our study of GD, a lysosomal storage disorder caused by biallelic mutations in the *GBA1* gene [18]. Patients with GD exhibit a near-complete deficiency in lysosomal enzyme GCase activity, leading to pronounced lysosomal dysfunction. In plasma-derived EVs from GD patients, we observed a shift toward larger vesicle populations compared to healthy controls detected by both cryo-EM and dynamic light scattering [18].

The association between *GBA1* mutations and an increased risk of PD is well established [12,13,14,15,16]. However, the precise molecular mechanisms underlying this connection remain incompletely understood. We and others have demonstrated that *GBA1* mutation carriers are characterized by reduced GCase activity and elevated levels of lysosphingolipids in blood and blood-derived cells [39,40,41,42,43,44,45,46,47]. These alterations, primarily occurring within lysosomes, may suggest a disturbance of sphingolipid metabolism that may affect vesicular trafficking, membrane dynamics, and EV biogenesis.

While reduced GCase activity is a common feature among *GBA1* mutation carriers, its link to PD risk and severity appears to be modulated by the type of mutation in the *GBA1* gene [43,44,45]. Some studies suggest that even modest decreases in GCase enzyme activity may contribute to PD pathogenesis, possibly through chronic lysosomal stress, while others highlight the importance of mutation-specific cellular effects [48,49,50]. However, the question is whether the heterozygous *GBA1* mutation in GBA1-PD, characterized by a moderate decrease in blood GCase activity and substrate accumulation, is sufficient to induce lysosomal dysfunction and thereby alter EV morphology.

First, we characterized the size and morphology of EVs isolated from the blood plasma of GBA1-PD patients stratified by mutation type (p.N370S, p.L444P), in comparison to healthy controls using cryo-EM. EVs can be isolated from various biological fluids, including plasma and CSF, and are characterized by specific protein markers [51]. While EV visualization is typically performed using transmission electron microscopy (TEM) or fluorescence microscopy, cryo-EM remains the most powerful technique for ultrastructural characterization, allowing the preservation of vesicle morphology in near-native conditions. Despite its advantages, only a few studies to date have employed cryo-EM to assess EV morphology in biological fluids [1,52,53,54,55,56,57]. In our study, cryo-EM analysis revealed an increase in EV size and substantial morphological heterogeneity in GBA1-PD samples. We identified single-membrane, double-membrane, multilayered, and electron-dense-cargo-containing vesicles. Notably, EVs from GBA1-PD patients showed a predominance of double- and multilayered structures, which were rare in controls. When stratified by mutation type, the largest EVs, as well as the largest size and the highest proportion of double and multilayered vesicles, were observed in PD patients carrying the p.N370S mutation. In contrast, EVs from p.L444P carriers displayed less pronounced size increases and fewer multilayered vesicles, suggesting that different *GBA1* mutations may differentially impact EV morphology, biogenesis, and structure. It is interesting to note that we previously described the increased number of EVs in GBA1-PD plasma and their increased size using the NTA technique [33].

Also, we report for the first time a comprehensive untargeted lipidomic analysis of plasma-derived EVs from patients with GBA1-PD, stratified by *GBA1* mutation type (p.N370S and p.L444P). Previous research has provided insights into the plasma lipidome of GBA1-PD patients, showing similarities between the lipid profiles of blood plasma from GD and GBA1-PD patients. For example, an increase in PC levels alongside a decrease in LPC was observed in the blood serum of both GD and GBA1-PD patients [58]. Additionally, a large study analyzing serum lipid levels in 415 PD patients—with and without *GBA1* mutations—reported elevated levels of monohexosylceramides, Cer and SMs in mutation carriers [59]. It is important to note, however, that this study did not include healthy controls in their analysis and did not distinguish between different *GBA1* mutations. Until now, only one study assessed the lipidome of EVs from GBA1-PD [60]. In this study, EVs were derived from post-mortem cerebrospinal fluid (CSF) of *GBA1* mutation carriers with PD, as well as with dementia and Lewy bodies. Lipidomic analyses demonstrated that EVs from *GBA1* carriers with synucleinopathies were loaded with ceramides, which is in agreement with our data. Kurzava-Akanbi et al. suggested that elevation of ceramides in EVs might be a mechanism of cells to dispose of excess lipids and demonstrated that EVs derived from GBA1-linked synucleinopathies induced alpha-synuclein aggregation in the system in vitro [60]. However, EV lipid composition was analyzed without stratification by mutation type. In contrast, our study provided a detailed comparison of EV lipid profiles between p.N370S and p.L444P carriers. Specifically, the p.L444P mutation is characterized by a pronounced increase in sphingolipids, particularly ceramides (Cer 23:1, Cer 24:0) and sphingomyelins, suggesting a mutation-specific dysregulation of sphingolipid metabolism. In contrast, patients carrying the p.N370S mutation exhibit broader lipidomic changes, encompassing significant alterations in various lipid classes, including TGs, PCs, phosphatidylethanolamines, and N-acylethanolamines, indicating a more extensive remodeling of the lipidome. These data suggest that while p.L444P carriers display a more focused sphingolipid imbalance, p.N370S carriers present a widespread disruption of lipid homeostasis affecting multiple lipid classes.

Previously, our study and those of other researchers demonstrated lysosomal dysfunction in dopaminergic neurons (DA neurons) derived from GBA1-PD patients carrying the p.N370S mutation. This dysfunction is characterized not only by decreased GCase activity and protein levels but also by alterations in the enzymatic activity of other lysosomal enzymes and increased concentrations of lysosphingolipids [61], as well as elevated lysosomal burden and dysregulation of the autophagy–lysosomal system. This includes impaired lysosomal degradation capacity, disrupted autophagic flux, and accumulation of undegraded material within lysosomal compartments, as shown by electron microscopy [62,63]. Additional evidence from fibroblast models indicates that the p.N370S mutation leads to ER retention of misfolded GCase, resulting in ER stress, structural disorganization, and fragmentation of the Golgi apparatus. These cells also exhibit excessive autophagosome accumulation, reflecting defective autophagic clearance, likely exacerbated by cholesterol accumulation and the formation of MLBs—membrane-rich lysosomal structures typically formed under lipid overload conditions [50]. Given the shared endolysosomal origin of MLBs and EVs, such as exosomes, the presence of MLBs may also impact EV biogenesis and cargo composition, particularly under lysosomal stress conditions. Consistent with these observations, our previous data from peripheral blood mononuclear cell (PBMC)-derived macrophages from p.N370S GBA1-PD patients further confirm that this mutation causes more pronounced disruption of the lysosomal–autophagic system than other *GBA1* variants [64]. Notably, such extensive lipid alterations in EVs of p.N370S carriers may stem directly from the more pronounced endolysosomal dysfunction described above.

In our current study, we observed elevated levels of long-chain ceramides (Cer 23:1, Cer 24:0), predominantly in patients carrying the p.L444P mutation, which supports the idea that these lipids may contribute to the mutation-specific pathogenesis of GBA1-PD. Recent data indicate that the accumulation of long-chain glycosphingolipids (≥C22), such as glucosylceramides, plays a critical role in inducing alpha-synuclein neuropathology in vivo. Specifically, ceramides with acyl chains longer than 22 carbons have been shown to stabilize neurotoxic forms of alpha-synuclein, thereby promoting its pathological aggregation [65]. Furthermore, in vitro studies demonstrated a pro-aggregative effect of lipids isolated from fibroblasts of GBA1-PD patients harboring the p.L444P mutation on alpha-synuclein fibril formation. The lipid composition of these fibril-derived mixtures primarily consisted of phosphatidylcholine (75% of total lipids), diacylglycerols (10%), and sphingolipids (12%), with the sphingolipid fraction composed of sphingomyelin, ceramide, and hexosylceramide [66]. This lipid profile closely resembles the lipid composition of exosome membranes, which are known to be enriched in these classes of lipids and play a role in exosome biogenesis [67]. The presence of similar lipid classes in plasma-derived EVs from GBA1-PD patients observed in the present study suggests their potential involvement in alpha-synuclein aggregate formation and propagation. It is plausible that in *GBA1* mutation carriers, lipids that accumulate due to impaired GCase activity are released via EVs, contributing to disease progression. Supporting this, recent findings have demonstrated that defective GCase activity in GBA1-PD fibroblasts promotes EV release independently of mutation severity. Notably, EVs derived from fibroblasts carrying severe p.L444P mutations induce increased phosphorylation of alpha-synuclein at Ser129 in neuronal cells compared to both healthy controls and carriers of milder p.N370S mutations [68]. Thus, the p.L444P mutation is not associated with pronounced lysosomal dysfunction as severe as that observed in p.N370S carriers. However, it may still contribute to alpha-synuclein accumulation due to its more pronounced impact on sphingolipid metabolism.

Altogether, these results highlight the distinct impact of *GBA1* mutation types on lipid dysregulation and EV-mediated alpha-synuclein pathology in PD.

Also, we assessed the protein content of plasma-derived EVs from patients with GBA1-PD. Interestingly, despite the relevance of alpha-synuclein in PD pathology, we did not detect alpha-synuclein protein in the EV protein cargo from GBA1-PD patients by MS/MS analysis. This finding aligns with previous proteomic analyses of plasma and serum exosomes from PD patients, which also failed to detect alpha-synuclein [30,69,70,71,72]. It is worth noting that identifying multiple protein targets in exosomes remains technically challenging, labor-intensive, costly, and limited by sample availability. Even MS/MS-based proteomic analyses may yield inconsistent results between samples, possibly due to technical variability in sample preparation. Previously, we and others demonstrated the similar alpha-synuclein levels in plasma and CSF EVs in GBA1-PD and controls by means of ELISA analyses [33,60]. Nevertheless, we identified significant disruptions in pathways involved in the negative regulation of lipid transport (GO:0032369), lipid localization (GO:1905953), endocytosis (GO:0045806), and receptor-mediated endocytosis (GO:0048261). These alterations may be directly related to the observed lipidomic imbalances, suggesting that the dysregulation of lipid composition in EVs impairs their proper trafficking and cellular uptake. Such impairments in lipid transport and vesicle internalization could contribute to defective lipid homeostasis and pathological lipid accumulation, which are critical factors in the pathogenesis of GBA1-PD.

**Figure 11:**
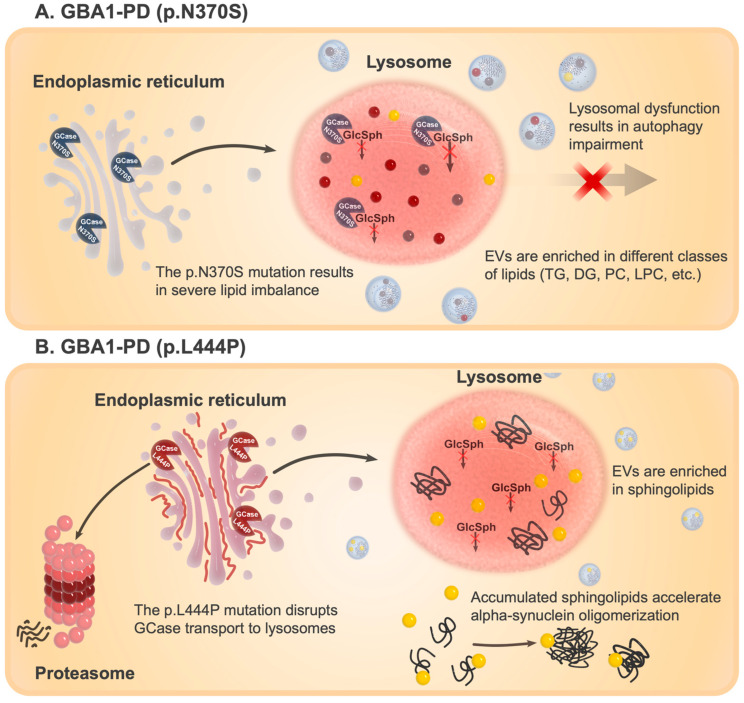
A schematic representation of the possible molecular mechanism behind the disruption of vesicle formation and changes in lipid and protein composition in GBA1-PD. (**A**) The EV profiles of individuals with the p.N370S mutation are characterized by larger vesicles, an increased presence of double- and multilayered structures, and notable changes in lipid composition—especially in sphingolipids, triglycerides, and cholesterol esters—as well as proteomic shifts associated with lipid transport and immune signaling. We suppose that the p.N370S mutation in the *GBA1* gene leads to more pronounced lysosomal dysfunction compared to p.L444P, likely due to its more efficient trafficking to lysosomes. Consequently, this results in a disruption of the endolysosomal system, affecting EV biogenesis, lipid sorting, and cargo release. (**B**) The p.L444P mutation causes early degradation of GCase, resulting in a more substantial reduction in enzyme activity and an accumulation of sphingolipids. Although this mutation does not cause severe disruptions in the endolysosomal pathway, it is believed to lead to a specific buildup of long-chain sphingolipids, including ceramides with acyl chains of C22 or longer. Created in Inkscape, version 1.3.2.

Consequently, the endolysosomal system becomes dysregulated, affecting EV biogenesis, lipid sorting, and cargo release. These alterations are reflected in the EV profile of p.N370S carriers, which show increased vesicle size, a higher proportion of double and multilayered structures, and extensive remodeling of lipid composition, particularly in sphingolipids, triglycerides, and cholesterol esters, along with proteomic changes linked to lipid transport and immune signaling. Based on our findings, we propose that p.N370S mutation in *GBA1* leads to more pronounced lysosomal dysfunction compared to p.L444P, likely due to its more efficient trafficking to lysosomes. Oppositely, p.L444P, due to its premature degradation in proteasomes, leads to a more significant reduction in GCase enzymatic activity and sphingolipid accumulation. Our results suggest that the p.L444P mutation, although it does not demonstrate such disturbances in the endolysosomal pathway, leads to a more focused accumulation of long-chain sphingolipids, including ceramides with acyl chains ≥ C22 (Figure 11). Supporting this, in vitro studies demonstrated that lipids isolated from fibroblasts of GBA1-PD patients carrying the p.L444P mutation enhance alpha-synuclein fibril formation, with EV membranes enriched in sphingolipids, ceramides known to mediate exosome biogenesis and cargo delivery. Moreover, our unpublished data indicate that the p.L444P mutation is associated with pronounced accumulation of alpha-synuclein in CD45^+^ blood cells. This observation is in line with other studies reporting elevated *SNCA* gene expression [73] and increased alpha-synuclein protein levels in p.L444P mutations compared to p.N370S on human neuroblastoma cell line BE(2)-M17 [49]. Altogether, these data support the hypothesis that EVs may mirror mutation-specific lysosomal dysfunction in GBA1-PD and play a role in disease propagation and heterogeneity, although the question of what exhibits the main effect, membrane lipids or membrane-associated proteins, remains. The altered lipid composition of GBA1-PD EVs identified in this study supports the notion that lipid EV cargo may be an inducer of alpha-synuclein fibrilization. It was shown that the interaction of alpha-synuclein with lipids is essential for the alpha-synuclein aggregation process [9,74,75].

The main limitation of our study is the relatively small sample size, which necessitated the use of pooled plasma EV samples for all analyses. Additionally, plasma EVs were not separated into specific subtypes based on tissue origin. For example, we did not isolate neuron-derived EVs, as has been performed in studies investigating lipid composition of plasma EVs in Alzheimer’s disease [76]. We isolated EVs using differential ultracentrifugation without additional purification to remove lipoproteins. Since plasma is lipoprotein-rich, their presence may affect lipidomic analysis. Moreover, while we performed proteomic analysis of EVs from GBA1-PD patients, we did not stratify the proteomic data according to mutation type (p.N370S vs. p.L444P), which may have masked mutation-specific protein signatures.

## 4. Materials and Methods

### 4.1. Participants

Blood samples from GBA1-PD patients (N = 11, average age 64.9 ± 10.8 years, 45% men, average age at onset 55.6 ± 11.2 years) and control group participants (N = 15, average age 62.5 ± 6.7 years, 48% men) were collected at the Pavlov First St. Petersburg State Medical University of St. Petersburg and N.P. Bechtereva Institute of the Human Brain, RAS, St. Petersburg. GBA1-PD patients—heterozygous carriers of common mutations in the *GBA1* gene (p.N370S and p.L444P)—were included in the present study. There were 5 patients with the p.N370S mutation and 6 patients with the p.L444P mutation. *GBA1* variants were confirmed in GBA1-PD patients and excluded in controls by genotyping, as previously described [16]. Plasma samples were isolated from peripheral blood using centrifugation at 3000 rpm and 4 °C for 20 min and stored at −70 °C. Samples were thawed on ice immediately before analysis. The study was conducted in accordance with the Declaration of Helsinki and approved by the Ethics Committee. Informed consent was obtained from all subjects involved in the study.

### 4.2. Isolation of EVs

EVs were isolated from equal volumes of plasma for each experiment (diluted 1:5 with PBS) after preliminary removal of cellular debris and large vesicles by centrifugation (2000× *g* for 30 min, and then 16,000× *g* for 30 min), and ultracentrifugation (Beckman Coulter centrifuge, Ti45 rotor) at 110,000× *g* for 2 h was performed. After centrifugation, the supernatant was removed, and the pellet was re-suspended in 0.5 mL of PBS for at least 1 h at 4 °C. Then 50 μL aliquots of the resuspended particles were taken for nanoparticle tracking analysis (NTA) and flow cytometry analysis. For proteomic, lipidomic analysis and cryo-EM EV preparations were mixed and re-centrifuged at 110,000× *g* for 2 h twice (SW 55Ti rotor). The resulting pellets were dissolved in 100 μL of PBS, aliquoted, rapidly frozen in liquid nitrogen, and stored at −80 °C until analysis.

### 4.3. Nanoparticle Tracking Analysis (NTA)

The size and concentration of EVs were determined by NTA using the NTA NanoSight1 LM10 (Malvern Instruments, Malvern, Worcestershire, UK) analyzer, equipped with a blue laser (45 mW at 488 nm) and a C11440-5B camera (Hamamatsu Photonics K.K., Iwata, Shizuoka, Japan). Recording and data analysis were performed using the NTA software 2.3. The following parameters were evaluated during analysis of the recording, monitored for 60 s: the average hydrodynamic diameter, the mode of distribution, the standard deviation, and the concentration of vesicles in the suspension. Before NTA measuring, an aliquot of the isolated vesicles was thawed at room temperature and diluted with deionized water in 10, 100, and 1000 times. The measurements were performed at least three times.

### 4.4. Flow Cytometry

Quantitative analysis of the exosomal markers (tetraspanins CD9 or CD81) on the surface of the isolated EVs was carried out using an ExoFACS ready-to-use kit for plasma exosome analysis (Lonza, Tallinn, Estonia) supplied with primary antibodies against CD9 and secondary Alexa488-labeled antibodies, according to the manufacturer’s recommendations. Additionally, bead-coupled EVs were assayed using an APC-conjugated Anti-CD81 antibody (Beckman Coulter, Brea, CA, USA). The same number of EVs was added to each sample for flow cytometry, based on the results of measurements of particle concentration using NTA. A sample without any EVs was used as a negative control for non-specific labeling. Analysis was performed with a CytoFlex instrument (Beckman Coulter, Brea, CA, USA).

### 4.5. Cryo-EM

Cryo-EM was used for direct visualization of EVs. To prepare samples for the cryo-EM study, lacey carbon EM grids were glow-discharged (30 s, 25 mA) in a Pelco EasiGlow system. An aliquot (3 μL) of the aqueous solution of the sample was applied on to the carbon side of the EM grid, which was then blotted for 2.0 s and plunge-frozen into the precooled liquid ethane with Vitrobot Mark IV (FEI, Hillsboro, OR, USA). This procedure results in embedding the samples in a thin layer of amorphous ice to preserve them in their native state and to protect them from radiation damage. The samples were studied in a cryo-electron microscope, Titan Krios 60–300 TEM/STEM (FEI, Hillsboro, OR, USA), equipped with a highly sensitive TEM direct electron detector (DED), Falcon II (FEI, Hillsboro, OR, USA), and a Cs image corrector (CEOS, Heidelberg, Germany) at an accelerating voltage of 300 kV. To minimize radiation damage during image acquisition, low-dose mode in EPU software, version 3.12 (FEI, Hillsboro, OR, USA) was used. Images were obtained with the Falcon II DED at 18,000× and 37,000× magnifications, with the defocus value in the range of [−2 μm; −5 μm]. The accumulated total dose per image did not exceed ~50 e^−^/Å^2^.

### 4.6. Western Blotting

The presence of the exosome-associated proteins (CD81 and CD63) in the samples of microvesicles was determined by Western blot. The ultracentrifugated pellets were incubated at 4 °C for 30 min with 20 μL of lysis buffer (7M urea, 2M thiourea, 4% CHAPS, 5 mM PMSF, 1% DTT). Total protein concentration was measured using a Pierce BSA Protein Assay kit (Thermo Scientific, Waltham, MA, USA). In total, 15 μg from each centrifugation pellet was loaded onto SDS-PAGE. Rabbit recombinant monoclonal CD81 antibody (ab109201, Abcam, Cambridge, UK) and rabbit polyclonal CD63 antibodies (ab216130, Abcam, Cambridge, UK) were used as primary antibodies in a dilution of 1:500. The goat anti-rabbit HRP conjugate (1:5000, ab6721, Abcam, Cambridge, UK) was used to detect both anti-CD81 and anti-CD63 antibodies. Digital images were obtained using the chemiluminescence system ChemiDoc (Bio-Rad, Hercules, CA, USA) and quantified using ImageJ software, version 1.52a.

### 4.7. Untargeted Lipidomic Analysis

The samples of EVs were stored at −80 °C until analysis. The samples were thawed on ice. Then, 200 μL of methanol and 750 μL of heptane containing the internal standard (SPLASH 330707) were added to the samples, which were then placed on an orbital shaker for 10 min at 10 °C. After incubation, the samples were homogenized for 2 cycles of 15 s, each at 5000 rpm. The samples were then centrifuged for 10 min at maximum speed. In total, 650 μL of the supernatant was taken from each sample and transferred to Eppendorf tubes. The samples were lyophilized to dryness and then reconstituted in 50 μL of phase B. The samples were centrifuged again, and 40 μL of the supernatant was taken, transferred to labeled vials with screw caps, and sent for analysis. The analysis was carried out using a Sciex 6600QTOF time-of-flight mass spectrometer (SCIEX, Framingham, MA, USA) with a calibrant delivery system (CDS) and an Exion 30AD liquid chromatograph (SCIEX, Framingham, MA, USA). Ion source settings: TEM = 400 °C; GS1 = 45; GS2 = 45; CUR = 35; and IS = 5500. Ion detection was performed in the positive ionization mode in TOFMS mode in the range of 400–1700 m/z. Chromatographic separation of the sample components was performed in RPLC mode using a Waters ACQUITY C8 2.1 × 100 mm 1.7 μm column: phase A (water:acetonitrile (4:6); 10 mM ammonium formate) and phase B (acetonitrile:2-propanol (1:9); 10 mM ammonium formate), with a 2 μL injection volume of the sample. The chromatographic gradient was as follows: 0 min 10% B; 0.25 min 10% B; 4 min 30% B; 5 min 48% B; 22 min 65% B; 24 min 99% B; 27 min 99% B; 27.5 min 10% B; and 30 min 10% B. The flow rate was 0.25 mL/min, the thermostat temperature was 55 °C, and the injection volume was 10 μL. SCIEX MasterView, Skyline, and MSDIAL software, version 24.1 were used for data processing.

### 4.8. Analysis of Lipidome Differential Expression of EVs

Lipid differential expression analyses of three groups were performed using the lipidr package in R (v.4.0.3). Lipidr provides statistical routines for determining differential expression in digital lipid expression data using a model based on negative binomial distribution [77]. The resulting *p*-values were adjusted using Benjamini and Hochberg’s approach for controlling the false discovery rate (FDR). Detected differential expression of lipids was considered statistically significant at *p* ≤ 0.05 and a fold change (FC) threshold of > 1.5. The differentially expressed lipids were visualized in a volcano plot built using EnhancedVolcano (v.3.3.3) in R (v.4.0.3). Lipid identifications with a signal-to-noise ratio > 5 and a signal intensity 5-fold higher than in the corresponding blank samples were considered for further data analysis as was performed earlier [66]. Lipid intensities were quantified as the peak areas of each lipid species, normalized using an internal standard to correct for technical variation. These normalized peak areas were used for all subsequent statistical analyses and comparisons. The full normalized lipidomic dataset is provided in Supplementary Table S5, containing all peak areas used for principal component analysis (PCA), differential expression testing, and other statistical analyses. PCA was performed on the normalized lipid abundances to assess global patterns of variation between samples. The PCA plots were generated using the plot_samples (d_normalized, “pca”) function in lipidr and are shown in Supplementary Figure S2, confirming the separation of experimental groups and the overall reproducibility of the lipidome data.

### 4.9. Untargeted Proteomic Analysis

Analysis of the exosome proteome was carried out at the Human Proteome Shared Use Center of the Research Institute of Biomedical Chemistry named after V.N. Orekhovich, using mass spectrometry HPLC-MS/MS with an Ultimate 3000 RSLCnano liquid chromatograph (Thermo Scientific, Waltham, MA, USA), connected to a Q-exactive HFX mass spectrometer (Thermo Scientific, Waltham, MA, USA). One microliter of the peptide mixture was loaded onto the enrichment Acclaim μ-Precolumn column (0.5 mm × 3 mm, particle size 5 μm, Thermo Scientific Waltham, MA, USA) at a flow rate of 10 µL/min for 4 min in isocratic mode using buffer “C” as the mobile phase (2% acetonitrile, 0.1% formic acid in deionized water). The peptides were then separated on an Acclaim Pepmap^®^ C18 HPLC column (75 µm × 150 mm, 2 µm particle size) (Thermo Scientific, Waltham, MA, USA) in gradient elution mode. The gradient was formed with mobile phase A (0.1% formic acid) and mobile phase B (80% acetonitrile, 0.1% aqueous solution of formic acid) at a flow rate of 0.3 µL/min. The column was washed with 2% mobile phase B for 10 min, after which the concentration of mobile phase B was linearly increased to 35% in 68 min and then linearly increased to 99% in 2 min. After a 2-min wash with 99% buffer B, the concentration of this buffer was linearly reduced to the original 2% in 3 min. The total duration of the analysis was 90 min. Mass spectrometric analysis was carried out on a Q-Exactive HFX mass spectrometer in positive ionization using a NESI source (Thermo Scientific, Waltham, MA, USA). The following parameters were set for mass spectrometric analysis: an emitter voltage of 2.1 kV and a capillary temperature of 240 °C. Panoramic scanning was carried out in the mass range from 300 m/z to 1500 m/z, with a resolution of 120,000. For tandem scanning, the resolution was set to 15,000 in the mass range from 100 m/z to the upper limit, which is determined automatically based on the mass precursor, but did not exceed 2000 m/z. Isolation of precursor ions was carried out in a window of ±1 Da. The maximum number of ions allowed for isolation in MS2 mode was set to no more than 40, with the cut-off limit for selecting a precursor for tandem mass analysis set to 50,000 units, and the normalized collision energy (NCE) was 29. For tandem scanning, only ions with z = 2+ to z = 6+ according to the charge state were considered. The maximum accumulation time for precursor ions was 50 ms, and for fragment ions, 110 ms. AGC values for precursors and fragment ions were set to 110^6^ and 210^5^, respectively. All measured precursors were dynamically excluded from tandem MS/MS analysis for 90 s. Protein identification was carried out using the SearchGUI v.3.3.20 program with simultaneous use of search algorithms: OMSSA and MS-GF+ [78,79]. The Human Proteome Uniprot database (UP000005640) was used to identify proteins. The following search parameters were specified: digestive enzyme—trypsin with the ability to skip two trypsin cleavage sites, accuracy of mass determination for monoisotopic peptides—±10 ppm, and accuracy of mass determination in MS/MS spectra—±0.05 Da. Carbamidomethylation of cysteine was taken into account as a mandatory modification of peptides. Oxidation of methionines, acetylation of lysines, phosphorylation of serine and threonine, guanidation of the N-terminus of the peptide, and the formation of propionamide on cysteine were taken into account as possible peptide modifications.

### 4.10. Analysis of Proteome Differential Expression of EVs

Statistical processing of data obtained during proteomic analysis was as follows. Over the course of the above-described proteomic studies, we used MaxQuant [80] software (version 2.7.3.0, https://www.maxquant.org/download_asset/maxquant/latest, accessed on 20 December 2024) and the library DEP package [81] (version 1.20.0, https://bioconductor.statistik.tu-dortmund.de/packages/3.16/bioc/manuals/DEP/man/DEP.pdf, accessed on 27 January 2025) in R (v.4.0.3) for the processing of a large array of mass spectrometric data. The resulting *p*-values were adjusted using Benjamini and Hochberg’s approach for controlling the false discovery rate (FDR). Detected differential expression of lipids was considered statistically significant at *p* ≤ 0.05 and a fold change (FC) threshold > 1.5. Then, to identify which biological processes, protein classes, and metabolic pathways were involved, the identified proteins were analyzed using online platforms for enrichment analysis, including KEGG MAPPER pathways (https://www.genome.jp/kegg/tool/map_pathway1.html, accessed on 21 February 2025). Additionally, Gene Ontology (GO) Biological Process enrichment was carried out using the ClueGO plugin within Cytoscape [82,83]. This allowed the visualization of enriched GO terms as functionally grouped networks. Enrichment significance was calculated using a two-sided hypergeometric test, with Bonferroni step-down correction applied for multiple testing. GO terms with adjusted *p* < 0.05 were considered significantly overrepresented.

### 4.11. Statistical Analysis

Data were analyzed using non-parametric methods due to non-normal distribution of variables. The Kruskal–Wallis test was used to assess differences among independent groups. When the Kruskal–Wallis test indicated significant differences (*p* < 0.05), and pairwise comparisons were performed using the Wilcoxon rank-sum test (Mann–Whitney U test) for independent samples. Fisher’s exact test was used for pairwise group comparisons to determine whether the morphology of EVs varied significantly across studied groups. To control for multiple comparisons, *p*-values were adjusted using the Bonferroni correction. All analyses were performed in R software (version 4.3.2).

## 5. Conclusions

In this study, we present the first comprehensive morphological, lipidomic, and proteomic characterization of blood plasma-derived EVs from GBA1-PD. Our data reveal that heterozygous *GBA1* mutation carriers exhibit altered EV biogenesis and lysosomal dysfunction, leading to increased EV size and structural complexity, particularly in p.N370S carriers. Lipidomic profiling uncovered significant remodeling of EV lipid composition, with mutation-specific enrichment patterns. p.N370S EVs showed widespread alterations across multiple lipid classes—including triglycerides, cholesteryl esters, ceramides, and phospholipids—while p.L444P EVs were predominantly enriched in sphingolipids. Proteomic analysis further identified dysregulation of lipid transport, immune pathways, and vesicle-mediated processes. These findings support the hypothesis that lipids carried by EVs to recipient cells—possibly neurons—may play a key role in GBA1-PD pathogenesis by affecting membrane composition, signaling, or metabolic stress responses.

## Data Availability

Data will be made available to interested researchers upon request.

## Supplementary Materials

The following supporting information can be downloaded at: https://www.mdpi.com/article/10.3390/ijms26189152/s1.

## Abbreviations

The following abbreviations are used in this manuscript:

CDS	Calibrant delivery system
CEs	Cholesteryl esters
Cer	Ceramides
Cryo-EM	Cryo-electron microscopy
CSF	Cerebrospinal fluid
DA neurons	Dopaminergic neurons
DED	Direct electron detector
DG	Diacylglycerols
EV	Extracellular vesicle
FC	Fold change
FDR	False discovery rate
GBA1-PD	GBA1-associated Parkinson’s disease
GCase	β-glucocerebrosidase
GD	Gaucher disease
HPLC-MS/MS	High-performance liquid chromatography–mass spectrometry
LPCs	Lysophosphatidylcholines
LSD	Lysosomal storage disorder
MGs	Monoacylglycerols
NAE	N-acylethanolamines
NCE	Normalized collision energy
NTA	Nanoparticle tracking analysis
PCA	Principal component analysis
PCs	Phosphatidylcholines
PD	Parkinson’s disease
PEs	Phosphatidylethanolamines
PGs	Phosphatidylglycerols
PI	Phosphatidylinositol
PS	Phosphatidylserine
SD	Standard deviation
SMs	Sphingomyelins
ST	Sterol
TEM	Transmission electron microscopy
TGs	Triacylglycerols
