# Supplementary material for: p.N370S GBA1 Mutation Influences the Morphology and Lipid Composition of Extracellular Vesicles in Blood Plasma from Patients with Parkinson’s Disease
Source: Int J Mol Sci. 2025 Sep 19;26(18):9152. doi: 10.3390/ijms26189152 (PMC12470647; doi:10.3390/ijms26189152)
Supplement: Supplementary file 1 [file ijms-26-09152-s001.zip › Table S2. Venn diagram analysis of differentially expressed lipids in blood plasma EVs from GBA1-PD patients and controls.pdf]

Table S2. Venn diagram analysis of differentially expressed lipids in blood plasma EVs from GBA1-PD patients and controls

Comparison Groups	Number of Lipids	Lipid Names
GBA1-PD (p.L444P) vs Controls, GBA1-PD (p.N370S) vs Controls, GBA1-PD (p.N370S) vs GBA1-PD (p.L444P)	11	CE 18:3, TG 56:1/TG 16:0_22:0_18:1, CE 18:1, CE 20:5, CE 22:6, Cer 41:1;O2/Cer 18:1;O2/23:0, CE 18:1(d7), TG 48:3/TG 16:1_16:1_16:1, TG 52:5/TG 16:1_18:2_18:2, SM 41:3;O2, TG 54:3/TG 18:1_18:1_18:1
GBA1-PD (p.L444P) vs Controls, GBA1-PD (p.N370S) vs Controls	3	SM 42:3;O2, TG 58:9/TG 18:1_18:2_22:6, TG 54:6/TG 16:0_18:2_20:4
GBA1-PD (p.N370S) vs Controls, GBA1-PD (p.N370S) vs GBA1-PD (p.L444P)	128	TG 54:1/TG 18:0_18:0_18:1 NAE 27:1 PE 38:6 TG 46:1/TG 14:0_16:0_16:1 NAE 26:6 SM 36:2;O2(d9)/SM 18:1;O2/18:1(d9) PC 35:1 PC 36:1/PC 18:0_18:1 PC O-37:0 DG 34:3 PG 33:1(d7)/PG 15:0_18:1(d7) DG 34:0/DG 16:0_18:0 PE 34:2 PC 38:2 PC 34:1/PC 16:0_18:1 DG 42:4 PC 36:2/PC 18:0_18:2 PC O-38:6/PC O-16:0_22:6 TG 48:1/TG 14:0_16:0_18:1 PE 38:4 PC 36:5/PC 16:0_20:5 DG 43:4 PE O-36:2 PC O-38:9 PC 36:0 PE 34:1/PE 16:0_18:1 PC O-34:1/PC O-18:1_16:0 PC 35:2 MG 22:0 PC 40:7 PC O-40:11 PC 35:4 PE P-40:5/PE P-18:0_22:5 TG 54:2/TG 18:0_18:1_18:1 TG 51:0/TG 16:0_17:0_18:0 TG 58:2/TG 16:0_24:0_18:2 DG 51:8 PC O-36:4 PC O-32:0/PC O-16:0_16:0 PC 38:4/PC 18:0_20:4 PC O-36:7 DG 44:3 DG 51:10 DG 47:13 PC 36:2 PC 34:0/PC 16:0_18:0 TG 60:2/TG 24:0_18:1_18:1 TG 51:1/TG 16:0_17:0_18:1 PC O-32:1 PC 40:6/PC 18:0_22:6 PC 37:7 PC O-35:7 DG 45:10 PE P-38:5/PE P-18:1_20:4 PC O-36:0 TG 55:3/TG 18:1_18:1_19:1 PC 36:4/PC 16:0_20:4 DG 34:0 DG 44:9 PC 40:4 TG 51:4/TG 15:0_18:2_18:2 TG 58:10/TG 18:2_18:2_22:6 DG 34:2/DG 16:0_18:2 NAE 9:0 PE P-40:6/PE P-18:0_22:6 PC 34:4 CE 18:2 TG 53:1/TG 17:0_18:0_18:1 DG 46:9 TG 52:2/TG 16:0_18:1_18:1 PS 38:4/PS 18:0_20:4 TG 54:7/TG 18:2_18:2_18:3 DG 36:0 SM 41:3;O3 PS 36:1 TG 51:2/TG 16:0_17:1_18:1 CE 20:4 TG 49:3/TG 15:0_16:1_18:2 TG 52:0/TG 16:0_18:0_18:0 PC 38:4 DG 46:4 DG 30:5 SM 34:0;O2 PC 32:0/PC 16:0_16:0 DG 36:0/DG 18:0_18:0 DG 37:7 DG 46:6 PC 40:5 TG 50:0/TG 16:0_16:0_18:0 PC O-38:5 TG 56:0/TG 16:0_16:0_24:0 PE P-38:6/PE P-16:0_22:6 TG 46:0/TG 14:0_16:0_16:0 PC 38:6/PC 16:0_22:6 NAE 10:0 TG 53:2/TG 17:0_18:1_18:1 PE P-36:4/PE P-16:0_20:4 TG 48:0/TG 16:0_16:0_16:0 ST 27:1;O NAE 20:1 TG 50:1/TG 16:0_16:0_18:1 DG 48:4 DG 33:1(d7)/DG 15:0_18:1(d7) TG 52:1/TG 16:0_18:0_18:1 PC O-34:4 DG 40:4 PE P-40:4/PE P-18:0_22:4 PE P-34:1/PE P-16:0_18:1 TG 48:1(d7)/TG 15:0_18:1(d7)_15:0 PE P-38:4/PE P-18:0_20:4 LPC 15:0 DG 40:2 PC 32:1 PC 34:2/PC 16:0_18:2 LPC 18:1(d7) TG 60:3/TG 24:0_18:1_18:2 PC O-35:4 PI 38:4 DG 44:4 TG 58:3/TG 16:0_24:1_18:2 PE O-35:2 TG 56:2/TG 20:0_18:1_18:1 TG 58:1/TG 16:0_24:0_18:1 TG 55:2/TG 19:0_18:1_18:1 DG 52:10 PE 36:2/PE 18:1_18:1 PC 34:2 PE 36:1
GBA1-PD (p.L444P) vs Controls, GBA1-PD (p.L444P) vs GBA1-PD (p.N370S)	4	TG 58:8/TG 18:1_18:1_22:6, SM 36:2;O2, SM 36:1;O2/SM 18:1;O2/18:0, LPC 16:0
GBA1-PD (p.N370S) vs Controls	44	SM 34:1;O2/SM 18:1;O2/16:0 PC 37:4/PC 17:0_20:4 PC 34:3 PC O-38:4 PC O-34:0/PC O-18:0_16:0 PC O-39:3/PC O-19:0_20:3 PC O-40:6 PE P-40:4/PE P-20:0_20:4 DG 36:3 TG 48:2/TG 14:0_16:0_18:2 PC 32:2 PE 40:5 SM 42:2;O2 DG 36:2/DG 18:1_18:1 PC 40:8/PC 20:4_20:4 TG 56:4/TG 18:1_20:1_18:2 TG 46:2/TG 12:0_16:0_18:2 SM 38:1;O2 TG 50:3/TG 14:0_18:1_18:2 TG 48:3/TG 14:0_16:1_18:2 TG 56:5/TG 18:0_18:1_20:4 TG 56:3/TG 18:1_18:1_20:1 PC 40:8 PC 33:1 PC 34:0 PC 36:4 TG 52:3/TG 16:0_18:1_18:2 PE 40:6 TG 54:0/TG 18:0_18:0_18:0 PE 40:4 TG 54:6/TG 18:2_18:2_18:2 SM 41:8;O2 TG 58:7/TG 18:1_18:1_22:5 TG 53:3/TG 17:0_18:1_18:2 TG 50:2/TG 16:0_16:1_18:1

Comparison Groups	Number of Lipids	Lipid Names
		LPC 18:0/0:0 PC O-33:2 Cer 40:1;O2/Cer 17:0;O2/23:1 PC 38:3/PC 18:0_20:3 TG 56:8/TG 16:0_18:2_22:6 PC O-38:5/PC O-18:1_20:4 TG 51:3/TG 15:0_18:1_18:2 TG 50:4/TG 16:1_16:1_18:2 PC 36:3/PC 16:0_20:3
GBA1-PD (p.L444P) vs Controls	6	Cer 42:1;O2/Cer 18:1;O2/24:0, SM 35:1;O2/SM 18:1;O2/17:0, SM 34:2;O2/SM 18:2;O2/16:0, TG 54:4/TG 18:1_18:1_18:2, CE 20:3, TG 56:7/TG 16:0_18:1_22:6
GBA1-PD (p.L444P) vs GBA1-PD (p.N370S)	10	SM 42:1;O2, CE 16:1, MG 18:0, SM 32:1;O2, PC 37:2, PS 40:6, DG 45:4, DG 50:10, TG 56:4/TG 18:0_18:0_20:4, DG 32:1/DG 16:0_16:1
