# Supplementary figures and images for: p.N370S GBA1 Mutation Influences the Morphology and Lipid Composition of Extracellular Vesicles in Blood Plasma from Patients with Parkinson’s Disease
Source: Int J Mol Sci. 2025 Sep 19;26(18):9152. doi: 10.3390/ijms26189152 (PMC12470647; doi:10.3390/ijms26189152)

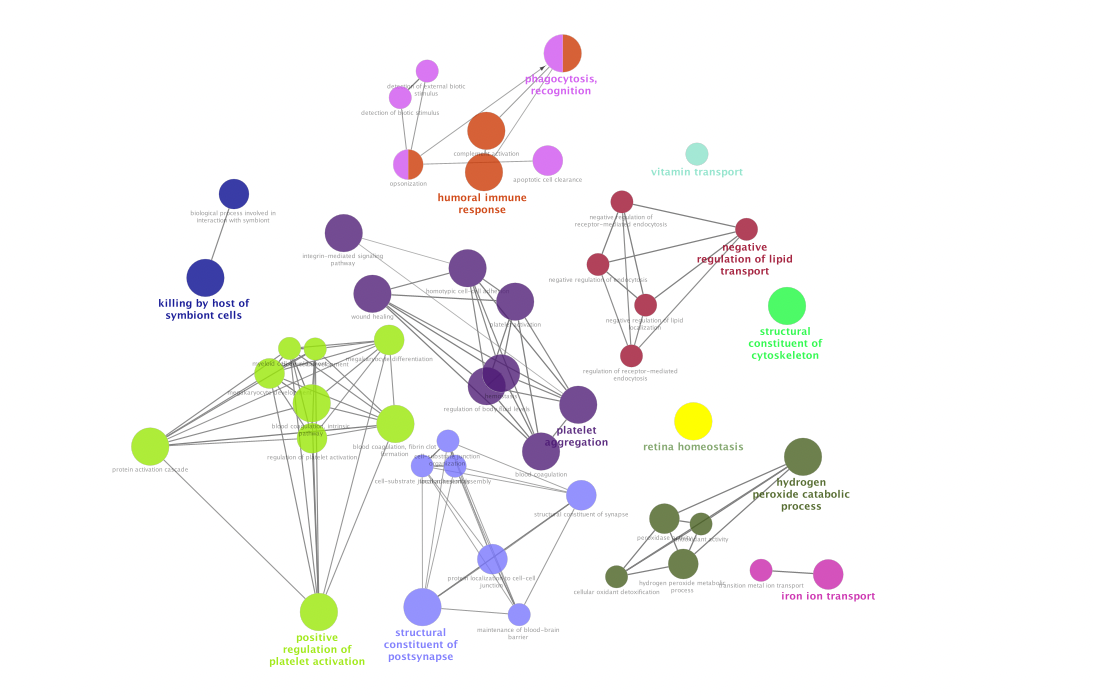

Supplement: Supplementary file 1 [file ijms-26-09152-s001.zip › Figure S1. Functional clusters derived from GO analysis of proteomic differences in blood plas-ma EVs between GBA1-PD patients and controls.png]

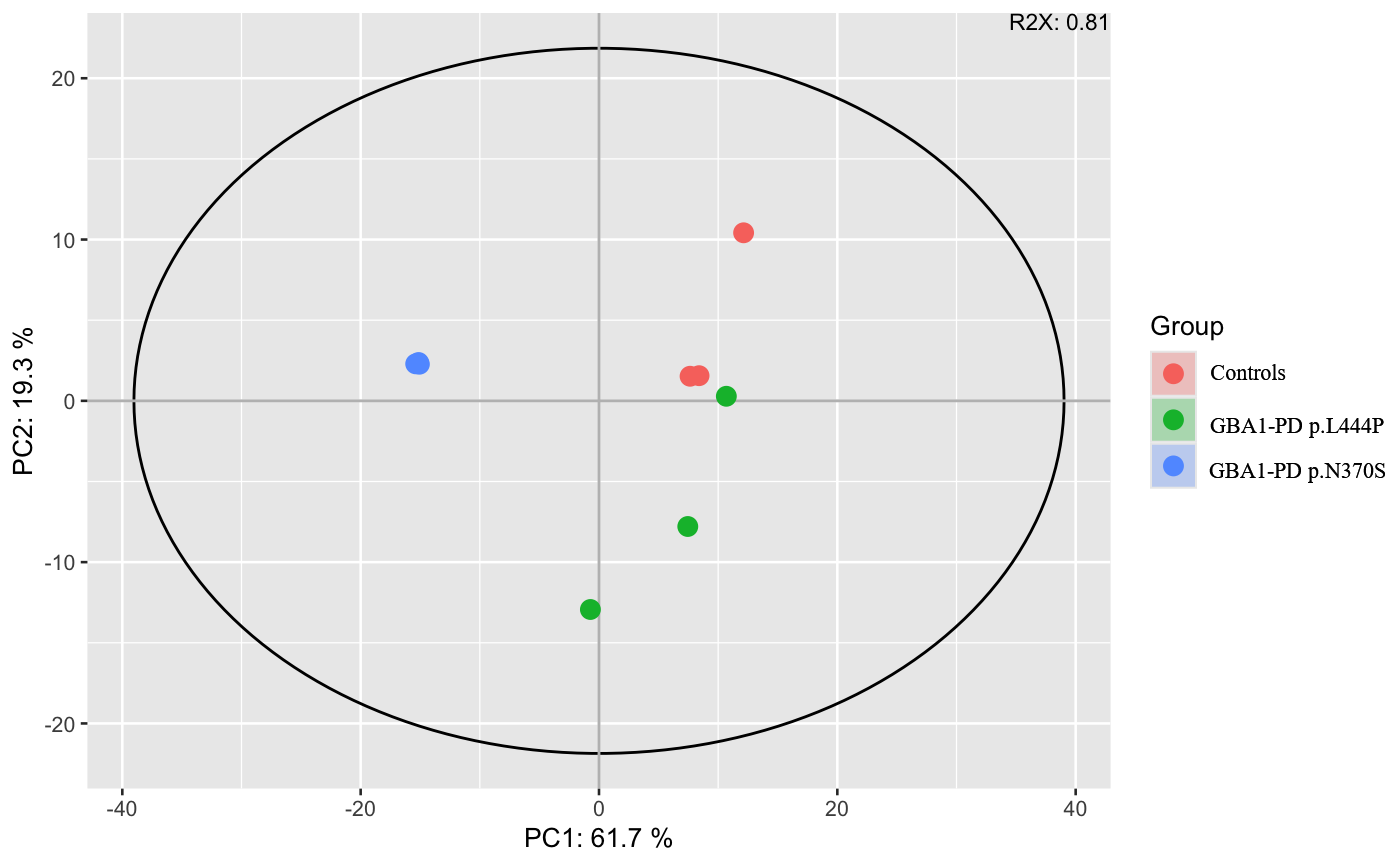

Supplement: Supplementary file 1 [file ijms-26-09152-s001.zip › Figure S2. Principal component analysis (PCA) of lipid profiles of blood plasma EVs from GBA1-PD patients and controls.tif]
